# The SIMCA Study Protocol: Factors influencing the implementation of the Midwifery Continuity of Carer (MCoC) model of care in NHS maternity care in England: A mixed methods cross case analysis involving clinicians, women and policy makers

**DOI:** 10.3310/nihropenres.13745.1

**Published:** 2025-01-16

**Authors:** Rebecca Milton, Susan Channon, Julia Sanders, Sara Kenyon, Aimee Middlemiss, Heather Strange, Kate Davies, Lena Choudary-Salter, Susan Barry, Tina Prendeville, Aled Jones

**Affiliations:** 1 Cardiff University Centre for Trials Research, Cardiff, Wales, UK; 2 Cardiff University School of Healthcare Sciences, Cardiff, Wales, UK; 3 School of Health Sciences, University of Birmingham, Birmingham, England, UK; 4 University of Plymouth School of Nursing and Midwifery, Plymouth, England, UK; 5 Tommy's Baby Charity, London, UK; 6 The Mosaic Community Trust, London, UK; 7 Imperial College Healthcare NHS Trust Division of Women's Children's and Clinical Support, London, England, UK; 8 Imperial College London Women's Health Research, London, UK

**Keywords:** Midwifery, maternity, continuity of carer, MCoC, continuity of care models, service delivery, patient safety, implementation.

## Abstract

**Background:**

During pregnancy, labour and early motherhood, most women in the UK receive care from different midwives. NHS policy change in England sought to introduce a model of care whereby each woman is cared for by the same midwife throughout antenatal, intrapartum and postnatal periods, supported by a small team of midwives to cover off-duty periods. This model is called the Midwifery Continuity of Carer (MCoC). This study proposes to evaluate the implementation and delivery of MCoC across England, aiming to better understand the factors that result in different rates of progress with MCoC implementation.

**Aim:**

To identify the local, regional and national factors which contribute to variable progress with implementation of MCoC in the NHS in England?

**Methods:**

A sequential mixed-methods study, informed by implementation science frameworks will be delivered over three work packages. Following a literature review of the challenges and successes of previous attempts to implement MCoC (work package 1), six case studies in NHS Trusts will be undertaken to better understand different rates of progress with MCoC implementation and people's experiences of MCoC implementation through: interview and questionnaire (maternity services staff); interviews (service-users); observation of relevant implementation meetings and organisational documentation collection (work package 2). Interviews will be undertaken with national and regional stakeholders relevant to MCoC implementation (work package 2). Data analysis will be conducted both inductively and deductively, informed by implementation science constructs (work package 3).

**Dissemination:**

Study findings will be disseminated through peer-reviewed journals, conferences and events. Results will be of interest to the public, clinical and policy stakeholders in the UK and will be disseminated accordingly.

## Plain Language Summary

Background

During pregnancy, labour, and early motherhood, most women in England receive care from different midwives. In 2016, NHS England introduced a policy aimed at ensuring that, by 2020, women would receive care from the same midwife (or a small team of midwives during off-duty periods) throughout their pregnancy and after birth. This model of care, called Midwifery Continuity of Carer (MCoC), has been introduced as there are strong claims that MCoC can improve the safety and quality of maternity care, especially for vulnerable women, babies, and those from minority ethnic communities or deprived areas. MCoC could also increase job satisfaction for midwives, although it might also lead to higher job-related stress and more unsociable working hours. While many midwives support the idea of MCoC, many also feel unable to implement it due to staffing shortages and other resource limitations, leading to mixed progress in England.

Aims

The study aims to understand the factors influencing the varied progress of MCoC implementation across England through three linked work packages (WPs).

Work packages

WP1: Conduct a literature review to understand the challenges and successes of previous MCoC implementation efforts.

WP2: Perform case studies in six NHS Trusts to explore different implementation rates and experiences. This includes interviews and surveys with maternity staff, service users, and stakeholders, as well as document reviews and observations of meeting.

WP3: Analyse data from the case studies to identify different approaches to MCoC implementation, including associated implementation factors, barriers and enablers, and patterns in MCoC outcomes.

Objectives:

- Review international literature on MCoC implementation challenges and successes.

- Evaluate how MCoC has been implemented and experienced in six diverse case sites.

- Explore the role of national and regional stakeholders in MCoC implementation.

- Synthesise findings to identify key implementation factors including barriers, facilitators and patterns in outcomes.

## Introduction

Improving newborn and maternal health has long been a leading priority of UK and global policy makers ^2, 3^. Yet safety and quality of maternity services remains problematic worldwide ^4^. Sub-optimal care in maternity services can result in death, serious disability and profound anguish for women, their children, and their families ^5, 6^, placing significant burden on healthcare systems and infrastructure, including the costs associated with legal action ^7^. The continuous and urgent need to enhance the quality and safety of care delivery is frequently linked to several factors, namely: multimorbidity, healthcare delivery complexities and a multitude of cultural and organisational challenges ^6, 8^.

As a result, recent NHS England policy has introduced significant changes to improve the quality and safety of maternity care ^1, 3^. Implementation of the policy for safer and more personalised care across England is currently led by the Maternity Transformation Programme (MTP), consisting of a range of inter-connected interventions, including establishing Midwifery Continuity of Carer (MCoC) models of care. MCoC aims to ensure that women are cared for by a named midwife who coordinates and personally provides the majority of care, supported by a small MCoC team (a headcount of eight midwives or fewer), throughout pregnancy, birth and the postnatal period, supported by a linked obstetrician ^9^. Although this evidence is still evolving and the extent of clinical advantages of the model have recently changed ^10^. However, little is known about the factors, contexts, and conditions necessary for successful implementation of policy initiatives to improve service delivery and care quality within the distinctive setting of maternity care ^11, 12^.

The question of implementing change in maternity services is particularly salient given the proliferation of priorities and initiatives introduced over the last five years within the ‘maternity and neonatal safety improvement programme’, coordinated by the MTP. Healthcare settings, which have similarly experienced a surfeit of interventions, have been described as ‘policy thickets’, which are defined as dense patches of overlapping goals that command substantial attention and resources, but where policy goals are unclear and external strategies may not link to local priorities ^13^. Policy thickets should be of particular interest to implementation research projects such as this. For example, important questions include how the implementation of each individual initiative interacts with other initiatives, such as MCoC implementation. Similarly, while each national initiative is generally well described whether they cumulatively stack-up as a coherent whole at the regional and local level, is often overlooked. The accumulation of local, regional and national maternity interventions also raises questions regarding the potential for MCoC implementation to be affected by ‘change fatigue’ within the workforce ^14^.

Questions relating to de-implementation are also relevant, such as how service leaders and other stakeholders plan and experience the redesign and decommissioning of existing services in response to new priorities. Potential difficulties and unintended consequences related to parallel and simultaneous implementation/de-implementation processes within clinical settings and teams are largely overlooked in existing research and policy ^15^.

Progress in implementing MCoC across England has been highly problematic ^16, 17^. Initial targets to deliver MCoC to the majority of women by 2021, with an interim target of 20% of women receiving MCoC by March 2019, were not met. For example, NHS statistics ^17^ indicated that in 2020, 108 Trusts offered MCoC to 15.9% of pregnant women, falling short of the interim target and significantly below the target of the ‘majority of women’. Implementation challenges were compounded by the COVID-19 pandemic, but this was not the only challenge with, a recent Health and Social Care Select Committee report rated the progress of MCoC implementation as highly variable and ‘requires improvement’ ^17^.

As a result, the implementation targets for MCoC have been regularly revised. Most recent amendments to MCoC implementation policy were issued in September 2022 ^18^. In response to the Ockenden report ^6^, NHS England directed all NHS Trust Chief Executives to ‘review and suspend, if necessary, the existing provision and further roll out of MCoC’ unless they could ‘demonstrate staffing meets safe minimum requirements on all shifts.’ Targets for implementation progress were also removed. Since autumn 2022 the MTP has maintained support for expansion of MCoC wherever possible. Some Trusts have successfully implemented MCoC, although the majority have partially implemented, or are yet to commence implementation. Progress with MCoC implementation is likely to remain variable for the foreseeable future, providing an opportunity to observe the challenges of implementation, as well as to describe the receptive context and the necessary conditions required for change.

### Rationale

Unproductive implementation in healthcare can cause workforce stress and uncertainty, especially if changes lack clear communication, fairness, or appropriate speed ^16, 19^. Failed efforts can overload staff, reducing patient care quality and treatment effectiveness. Implementing change in the NHS ^20, 21^, particularly in maternity services ^4, 22^, is challenging. Studying the implementation of MCoC within this context, amidst various initiatives and operational hurdles, is crucial. While limited research exists on MCoC implementation in NHS England, early evidence indicates complexity and challenges. A recent Cochrane review suggests future research should focus on understanding the implementation and scaling up of midwife continuity of care ^10^.

Evaluating local, regional and national factors relevant to MCoC implementation will inform policy discussions and improve decision-making in maternity settings in England and beyond. The aim is to explore factors influencing MCoC implementation in England, examining variations in operationalisation, sustainability, and experience. The research question is: "What factors at local, regional and national levels contribute to variable progress in MCoC implementation in NHS England?"

## Protocol

### Objectives

1. Appraise the international literature to understand the factors contributing to the success and challenges of MCoC implementation.

2. Evaluate how implementation decisions have been operationalised, sustained and experienced in six case study sites representing contrasting progress with MCoC implementation.

3. Describe and explore the role played by national and regional stakeholders in MCoC implementation.

4. Synthesise findings to identify various approaches to MCoC implementation, key implementation factors and relationships, and any discernible patterns between implementation factors and routinely reported MCoC outcomes.

### Theoretical/conceptual framework

MCoC is conceptualised as a complex intervention, e.g. one that comprises many inter-dependent components across multiple systems from the macro level (e.g. NHS England), to meso (e.g. Regional Midwifery Boards) and micro levels (e.g. Local Maternity Services) ^23^. These complex organisational levels are ‘nested’ ^24^, such that each level simultaneously interacts with multiple other systems. For example, MCoC implementation will occur alongside pre-existing micro-level employee relationships and experiences, as well as the characteristics of the maternity unit (e.g., size and setting). A range of contextual and organisational preconditions also exist at the meso-level, such as organisational/managerial structures, policies, processes, and hierarchies, which can shape the local implementation. In addition, public, policy and governmental interest in MCoC adds a social and inter-institutional macro level dimension to the implementation, which may be experienced from an institutional standpoint as external social and policy pressure and risk ^25^.

Given the complex nature of MCoC implementation and the contexts within which the intervention is being implemented, Normalisation Process Theory (NPT) ^26, 27^ and the Consolidated Framework for Implementation Research (CFIR) ^25^ offer appropriate and complementary frameworks to guide the study. NPT and CFIR are often used in combination with other theories to explore multiple facets of implementation ^27, 28^.

CFIR is not intended to be applied wholesale, but rather offers numerous constructs to consider when investigating implementation of complex interventions ^28^. In particular, CFIR constructs focussing on the interaction between the inner and outer settings within which an intervention is implemented are useful, given the complexity and national profile of MCoC. Generally, the outer setting includes the wider national/regional economic, political, and social context within which an organisation resides, and the inner setting includes features of local organisations’ structural, political, and cultural contexts through which the implementation process proceeds ^25^.

NPT seeks to explain how complex interventions work by focusing on factors promoting and inhibiting their transformation into routine ways of working ^29^. NPT consists of four main components, or generative mechanisms: coherence, cognitive participation, collective action and reflexive monitoring ^26^.

## Methods

### Patient and Public Involvement

Since study inception there has been active involvement and engagement from patient and public members. The Patient and Public Involvement (PPI) members of SIMCA are named co-applicants on the grant and have contributed to the development of the study. There is an established PPI group which meets regularly, similarly the PPI members participate as full members of the monthly Study Management Group (SMG). The Project Advisory Group (PAG) which meets six monthly has a PPI representative as a core member. The aim of this active engagement from inception is to ensure that patient and public views are integrated throughout the lifetime of the project as well to help the research team take a broader look at the context of maternity services, trying to understand the wider system of healthcare (e.g., the interface of maternity services and primary care), how national and regional decisions and systems reflect the needs of communities and individuals, and how these might impact on MCoC.

PPI members will focus on ensuring that the study is appropriately designed and delivered; e.g. contributing to developing the analysis, exploring findings and dissemination from a public/patient perspective. 

PPI members will also contribute directly to dissemination. Dissemination will have significant public reach through the close involvement in the project of Tommy’s Baby Charity and The Mosaic Community Trust.

Preparation of research information will include input from our PPI team, to ensure culturally appropriate content is distributed. Similarly, the PPI co-applicants will provide cultural sensitivity and awareness training to all members of the research team as specialist input for those undertaking interviews with women.

### Work packages

The project consists of the three inter-related work packages (WPs).

***Work Package 1: Narrative evidence synthesis***

The aim of WP1 is to undertake a narrative evidence synthesis approach which addresses objective 1. We will use a textual approach to generate an interpretive synthesis of any relevant ‘theories of change’ ^30^, contextual factors and organisational mechanisms that influence (for better or worse) the implementation of MCoC.

Results of selected studies will be gathered into a framework informed by CFIR constructs and supplemented by NPT (such as the focus on internal and external implementation factors). The framework approach ensures that the review focusses on the factors influencing implementation of MCoC, rather than reviewing the results of MCoC interventions per se. The final stage of WP1 will produce a synthesis of the results which will directly inform all subsequent work packages. 

***Work package 2: Comparative case studies and national and regional stakeholder interviews***

WP2 addresses research objectives 2 and 3. Comparative case study methodology will be used to facilitate the in-depth exploration of complex organisations, such as maternity services. This is achieved through combining a range of data collection methods, including surveys, interviews, observations and documents, with a variety of sampling techniques, to gain an in-depth understanding of the implementation factors and processes within each study site ^31^ as well as explore the perspectives of key national and regional stakeholders on the implementation of MCoC. 

### Study setting

WP2 will be conducted across six NHS settings in England.

### Study participants

We aim to conduct up to 65 national and regional stakeholder interviews and 90 participant interviews across the six case sites; 15 interviews per site (n=10) purposively sampled participants including those directly involved in MCoC implementation, for example, managers, midwives, obstetricians and (n=5) women enrolled in MCoC.

### Sampling and selecting national and regional stakeholders

For the purposes of this study, we define stakeholders as individuals and/or organisations who directly affect, or are affected by, MCoC implementation. National and regional stakeholders can have considerable influence over MCoC implementation by directly controlling resources and informing/taking key decisions. Individuals will be purposively sampled to recruit respondents with knowledge of MCoC, and/or involved in policy/strategy implementation. Potential participants include those contributing to MCoC and MTP implementation nationally within NHS (E/I) and NHS Health Education England (HEE). Stakeholders will be identified, contacted and recruited via accessing publicly available information from professional bodies (e.g. Royal Colleges), third sector organisations (e.g. Maternity Action), national and regional NHS representative bodies and national maternity voices programme (who support the co-production of maternity and neonatal services with service-users) for example. Regional NHS stakeholders will be geographically linked to the location of each case site and are likely to include representatives from regional maternity boards and regional MCoC and workforce planning leads. The research team’s extensive existing networks will also be utilised and referral from those contacted or recruited using the above methods.

### Data collection and management

Recorded semi-structured interviews with regional and national leads (up to 65): Candidate questions and interview schedules will be prepared as outlined above for case study interviews, with a particular focus on regional and national decision-making, implementation and de-implementation strategies and boundary working with local maternity settings. All participants taking part in interviews will be offered the choice of online applications (e.g., MS Teams) or face-to-face and recorded with permission. Interviews will be transcribed in full by an authorised external transcription company.

### Sampling and selecting case studies

Six case study sites will be selected following further examination of NHS England (NHSE) MCoC implementation data and discussion with key MCoC implementation leads at NHSE. The purposive sampling strategy will be informed by:

- Consideration of the regional and geographical settings of case study sites to ensure that case studies are undertaken in different regions of England and in rural, urban, and inner-city areas.
- Identifying ‘positive deviants’, defined as ‘organisations, teams or individuals that a consistently demonstrate high performance in an area of interest’ ^32^.

Positive deviance will be identified in a range of ways, including reviewing NHSE data on Trusts who have a high percentage of women placed on MCoC pathway by 28 weeks’ gestation. We will also incorporate a more rounded conception of positive deviance, by looking beyond outcome data produced by Trusts. For example, we will not discount the possibility that local pockets of high performance can also exist in Trusts that may have lower percentage of women placed on the MCoC pathway.

### Data collection and management

Access to undertake fieldwork in the case study sites will be negotiated with local stakeholders. In each case study, data will be generated via:

Observations: researchers will undertake guided non-participant observations at MCoC implementation meetings and related activities at each case site.

Local documentation and data: The researchers will access local documents via the stakeholders. These may include:

- Routinely collected MCoC implementation data.
- Anonymised patient safety data (e.g. serious incidents and events reports, staff concerns via Speak Up Guardians).
- Local documents (for example, MCoC operational policies and service specifications).
- MCoC service use.
- Completed local audits and/or evaluations.
- Related grey literature.

Staff survey: the survey will be used to collect the perceptions and experiences of maternity staff about the implementation of MCoC in the maternity services within which they work.

Descriptive analysis of the survey responses will initially explore how answers are distributed. In line with the guidance provided by the tool’s creators ^33^, total scores for the survey will not be calculated. Cronbach α testing will be conducted on all four NPT components, to measure the internal consistency of the constructs within the context of this study. Each NPT component will be derived as the mean score of the four questions in the survey that correspond to that NPT component. Components will then be summarised and examined for potential associations by various roles or organisational characteristics. Descriptive statistics and bar charts will help visualise the ‘shape’ of the data within and eventually across case sites. These steps will help identify interesting or anomalous features within the data and prove useful in then generating cross-tabulations and scattergrams of the relationships between implementation factors and other variables. Survey analysis will be undertaken via SPSS.

Recorded semi-structured interviews in six case study sites (n=c.90): At each case site semi-structured interviews (n=15) will be conducted with purposively sampled participants including those directly involved in MCoC implementation, for example, managers, midwives, obstetricians (n=10) and women enrolled in MCoC (n=5).

Interview schedules will be informed by the staff survey findings, in addition to views of the PAG and PPI team, the findings of the narrative synthesis and the application of CFIR and NPT via their respective toolkits ^34, 35^. Questions will be included on:

- How services are organised and delivered.
- Any effect on implementation of the interplay between the ‘outer domain’ (regional and national priorities and incentives) and the ‘inner domain’ (maternity services).
- Organisational readiness and the ‘implementation climate’ related to MCoC.
- The coherence of MCoC implementation to staff and women.
- Resources allocated to embedding and sustaining the MCoC model of care.
- The effect of MCoC on other maternity services and how existing services are decommissioned/de-implemented.

All participants taking part in interviews will be offered the choice of online applications (e.g., MS Teams) or face-to-face and recorded with permission. Interviews will be transcribed in full by an authorised external transcription company.

### Data analysis

Thematic analysis of qualitative data sources, underpinned by methodological rigour ^35^, will be undertaken by the core project team, concurrent with data collection in each case site. NPT and CFIR constructs will iteratively inform each step of the analysis to provide rich understanding of the operational context and implementation of MCoC. Separate analysis of each case study and the regional and national stakeholder interviews will commence with data familiarisation, initial inductive and theoretical coding drawing on findings from WP1, and the identification of themes. All analysis will be overseen by a senior researcher. Other members of the team, including the PPI members, will also periodically review transcripts to ensure consistency and contribute to analysis via online and face-to-face team meetings.

The combined WP2 analytic process will involve:

- Using the latest version of the NVivo qualitative data analysis software ( https://lumivero.com/products/nvivo/) and SPSS ( www.ibm.com/spss) for the survey data to organise and store data ready for analysis.
- In-depth and iterative familiarisation of interview transcripts and field-notes followed by inductive thematic analysis ^35^. The analysis will identify a range of respondents’ views including local (micro level), regional (meso level) and national (macro level) participants.
- Methodological rigour will be ensured through standard procedures of reflexivity ^36^. Regular analysis meetings will be held within and between the teams in Cardiff University and University of Plymouth.
- Convergent analysis ^31^, via triangulation of the quantitative (survey) and qualitative datasets, will establish patterns of within-case similarities and differences regarding MCoC implementation.
- A comparative, cross-case synthesis will then follow in WP3 (see below), though we have also scheduled a period into the WP2 timeline to explicitly plan and prepare for our transition from within-case to cross-case analysis.

***Work Package 3: Cross-case analysis and synthesis of findings***

WP3 addresses objective 4. This objective will be achieved by comparing and contrasting factors influential to each case study’s approach to the development, organisation, and implementation of the MCoC model of care. The process of cross-case analysis and synthesis will follow a matrix approach ^37^, consisting of a ‘tabular format that collects and arranges data for easy viewing in one place and permits cross-case analysis’. Specifically, to integrate findings across cases an inductive ‘data condensation’ process, foreshadowed by the overall research question and objectives, will initially be used to select, focus and simplify relevant findings from each site. Extracted findings will populate a series of cross-case thematic tables informed by NPT and CFIR frameworks, in order to map and understand the range of views and experiences across sites. Local implementation decisions will also be considered alongside the findings of the national and regional stakeholder interviews and the findings of the WP1 narrative synthesis of MCoC implementation.

Table 1 presents an overview of protocol and study related information. Table 2 displays the protocol amendments to date.

**Table 1.** Dataset.

Data category	Information
Primary registry and trial identifying number	ISRCTN10635039
Date of registration in primary registry	18 ^th^ March 2024
Source(s) of monetary or material support	NIHR Health and Social Care Delivery Research
Primary sponsor	University of Plymouth
Contact for public queries	simca@cardiff.ac.uk
Contact for scientific queries	simca@cardiff.ac.uk / aled.jones@plymouth.ac.uk
Public title	Factors influencing the implementation of the Midwifery Continuity of Care (MCoC) model of care in England: A mixed methods cross case analysis
Countries of recruitment	England
Health condition(s) or problem(s) studied	Implementation of the Midwifery Continuity of Care (MCoC)
Intervention(s)	N/A
Key inclusion and exclusion criteria	Inclusion criteria: - Individuals who directly affect, or are affected by, MCoC implementation. - Are associated with a case site. Exclusion criteria: - No groups are to be excluded from participating, unless there are clinical grounds barring participation following discussion with the midwifery team.
Study type	A mixed methods cross case analysis
Date of first enrolment	19/07/2023
Target sample size	90 (semi-structured interviews)
Recruitment status	Open
Primary outcome(s)	The main outcomes of the study will be to identify various local, regional and national approaches to MCoC implementation and key implementation factors and relationships and any discernible patterns between implementation factors and routinely reported MCoC outcomes. Through better understanding of local, regional and national factors contributing to varying progress with MCoC implementation, the findings of the study can be used to inform ongoing implementation of MCoC in England, and elsewhere and contribute to debates about future changes to maternity services.

**Table 2.** Protocol amendments.

Amendment No.	Protocol version no.	Date issued	Summary of changes made since previous version
1	1.1	29/08/23	Comments and suggestions implemented from University of Plymouth’s review process.
2	1.2	28/09/23	Addition of recruitment details for service users in section 9.1. Updated milestones table (section 21) to reflect study set up delays.
3	1.3	10/10/23	Add website to page 1. Added in statement re. UoP granting ethical approval (section 17.1). Updated Appendices section with new file names.
4	2.0	11/12/23	Change to section 9.1 in relation to members of the clinical team approaching service-users obtaining consent to contact.
5	3.0	17/04/2024	Addition of ISRCTN number. Removal of development of data management plan. Addition of incentive for service user interviews Updated section 23
6	4.0	19/06/2024	Reduce the number of case study sites from nine to six and alter subsequent sample sizes.

## Ethics and consent

Throughout this study we will follow the principles of good practice set out in the UK Policy Framework for Health and Social Care Research (Health Research Authority *et al.*, 2021). Ethical issues in this project arise in WP2: Comparative Case Studies and National and Regional Stakeholder Interviews. The primary ethical and research governance issues here are consent, anonymity, confidentiality, data protection and the safety of participants and researchers. Regarding consent, we will follow standard ethical procedures for gaining written informed consent from participants prior to them participating in the interview and subsequent them reading and considering the participant information sheets. In relation to data protection, all data we collect will be confidential to the project and stored securely in line with current University and NHS research governance and general data protection regulations. Any identifiable data will be anonymised prior to analysis in line with good research practice. In the context of participant safety and wellbeing, researchers will be trained in good interview practice as well as the use of distress protocols (including immediately ceasing the interview if participants become upset and providing avenues for support) and a disclosure protocol. All researchers accessing participants will be DBS checked. Regarding researcher safety, we will follow the relevant University’s lone working policy.

This study protocol has been approved by NHS, East Midlands – Nottingham 2 Research Ethics Committee and Health Research Authority, REC reference 23/EM/0272, approval date 14 ^th^ December 2023. The national and regional stakeholder interviews were approved by University of Plymouth Faculty Research Ethics and Integrity Committee, approval date was 24 ^th^ March 2023.

## Dissemination

Dissemination will occur throughout the project. Insights will contribute to current and future implementation of complex initiatives within maternity and other NHS services. Dissemination outputs will include clear, actionable, lessons to advance implementation decision making of national, regional, and local policy makers and practitioners. Findings will also be disseminated via international peer reviewed journals and conferences. PPI is embedded into each WP and a range of public engagement and dissemination events are planned throughout the project’s duration. The report will follow the NIHR threaded publication format. Project report and papers will be produced detailing findings and recommendations, training materials to be developed for use in other maternity services and in other NHS services. Results will be of interest to clinicians, practitioners and policy makers in the UK.

## Data Availability

No data are associated with this article. Figshare: SIMCA Study Material, Doi: https://doi.org/10.6084/m9.figshare.27831345.v1 ^38^ *This project contains the following extended data:* 20230317SIMCAStakeholdersConsentFormONLINEv2_0.pdf SIMCA CASE SITE INTERVIEW GUIDE Board level.docx SIMCA CASE SITE INTERVIEW GUIDE Midwifery management.docx SIMCA CASE SITE INTERVIEW GUIDE Midwives.docx SIMCA CASE SITE INTERVIEW GUIDE Women and other service users.docx SIMCA Consent Form - Service Providers - v1.2 240124.docx SIMCA Consent Form - Service Users - v1.3 17042024.pdf SIMCA Participant information sheet - Service Providers - v2.1 240124.pdf SIMCA Participant information sheet - Service Users - v3.0 17042024.pdf SIMCA PIS - Stakeholders v3.0 17032023.docx SIMCA Poster - Service Providers - v1.1 240124.pdf SIMCA Poster - Service Users - v3.0 170424.pptx SIMCA STAKEHOLDER INTERVIEW GUIDE - National regional midwives.docx SIMCA STAKEHOLDER INTERVIEW GUIDE – NHSE.docx SIMCA STAKEHOLDER INTERVIEW GUIDE - Service user orgs and reps.docx Data is available under the terms of the CC BY 4.0 Figshare: SPIRIT reporting guidelines ^39, 40^ “The SIMCA Study Protocol: Factors influencing the implementation of the Midwifery Continuity of Carer (MCoC) model of Care in England: A mixed methods cross case analysis.” Doi: https://doi.org/10.6084/m9.figshare.27831891.v1. Data is available under the terms of the CC BY 4.0

## References

[1] England N: Better Births. Improving outcomes of maternity services in England. A five year forward view for maternity care.2016. Reference Source

[2] World Health Organization: Recommendations on Maternal Health.2017. Reference Source

[3] NHS: The Long Term Plan.2019. Reference Source

[4] Liberati EG Tarrant C Willars J : Seven features of safety in maternity units: a framework based on multisite ethnography and stakeholder consultation. *BMJ Qual Saf.* 2021;30(6):444–456. 10.1136/bmjqs-2020-010988 32978322 PMC8142434

[5] Andreasen S Backe B Jørstad RG : A nationwide descriptive study of obstetric claims for compensation in Norway. *Acta Obstet Gynecol Scand.* 2012;91(10):1191–5. 10.1111/j.1600-0412.2012.01409.x 22486308

[6] Independent Review of Maternity Services at The Shrewsbury and Telford Hospital NHS Trust (Great Britain), Ockenden D, Great Britain: Ockenden report - Final: return to an address of the honourable the house of commons dated 30 March 2022 for Findings, Conclusions and Essential actions from the Independent Review of Maternity Services at the Shrewsbury and Telford Hospital NHS Trust: our final report. Department of Health & Social Care,250. Reference Source

[7] Magro M : Five years of cerebral palsy claims: NHS Resolution a thematic review of NHS Resolution.2017. Reference Source

[8] Royal College of Obstetricians & Gynaecologists: Royal College of Obstetricians & Gynaecologists.workforce report 2022,2022. Reference Source

[9] NHS England: Delivering Midwifery Continuity of Carer at full scale.2021; [cited 2023 May 30]. Reference Source

[10] Sandall J Fernandez Turienzo C Devane D : Midwife continuity of care models versus other models of care for childbearing women. *Cochrane Database Syst Rev.* 2024;4(4): CD004667. 10.1002/14651858.CD004667.pub6 38597126 PMC11005019

[11] Medley N Vogel JP Care A : Interventions during pregnancy to prevent preterm birth: an overview of Cochrane systematic reviews. *Cochrane Database Syst Rev.* John Wiley and Sons Ltd;2018;11(11): CD012505. 10.1002/14651858.CD012505.pub2 30480756 PMC6516886

[12] Fernandez Turienzo C Rayment-Jones H Roe Y : A realist review to explore how midwifery continuity of care may influence preterm birth in pregnant women. *Birth.* 2021;48(3):375–88. 10.1111/birt.12547 33749001

[13] Dixon-Woods M Baker R Charles K : Culture and behaviour in the English National Health Service overview of lessons from a large multimethod study. *BMJ Qual Saf.* 2014;23(2):106–15. 10.1136/bmjqs-2013-001947 24019507 PMC3913222

[14] Taylor B Hewison A Cross-Sudworth F : Transformational Change in maternity services in England: a longitudinal qualitative study of a national transformation programme ‘Early Adopter’. *BMC Health Serv Res.* 2022;22(1): 57. 10.1186/s12913-021-07375-3 35022052 PMC8753811

[15] Williams I Harlock J Robert G : Is the end in sight? A study of how and why services are decommissioned in the English National Health Service. *Sociol Health Illn.* 2021;43(2):441–458. 10.1111/1467-9566.13234 33636017

[16] Royal College of Midwives: The RCM’s stance on continuity of carer. Midwives,2021;24.

[17] Social Care Committee: The Health and Social Care Committee’s Expert Panel: evaluation of the Government’s progress against its policy commitments in the area of maternity services in England First Special Report of Session 2021–22.2021. Reference Source

[18] NHS England: B2011 Midwifery Continuity of Carer letter.2022; [cited 2024 Jul 11]. Reference Source

[19] Nilsen P Schildmeijer K Ericsson C : Implementation of change in health care in Sweden: a qualitative study of professionals’ change responses. *Implement Sci.* 2019;14(1): 51. 10.1186/s13012-019-0902-6 31088483 PMC6518624

[20] Dixon J : Improving the quality of care in health systems: towards better strategies. *Isr J Health Policy Res.* 2021;10(1): 15. 10.1186/s13584-021-00448-y 33608042 PMC7893377

[21] Marshall M Davies H Ward V : Optimising the impact of health services research on the organisation and delivery of health services: a mixed-methods study. *Health Soc Care Deliv Res.* 2022;10(3). 10.3310/HFUU3193 35157415

[22] Liberati E Tarrant C Willars J : How to be a very safe maternity unit: an ethnographic study. *Soc Sci Med.* 2019;223:64–72. 10.1016/j.socscimed.2019.01.035 30710763 PMC6391593

[23] Skivington K Matthews L Simpson SA : Framework for the development and evaluation of complex interventions: gap analysis, workshop and consultation-informed update. *Health Technol Assess.* 2021;25(57):1–132. 10.3310/hta25570 34590577 PMC7614019

[24] Hannigan B : Connections and consequences in complex systems: insights from a case study of the emergence and local impact of crisis resolution and home treatment services. *Soc Sci Med.* 2013;93:212–9. 10.1016/j.socscimed.2011.12.044 22386638

[25] Damschroder LJ Reardon CM Widerquist MAO : The updated Consolidated Framework for Implementation Research based on user feedback. *Implement Sci.* 2022;17(1): 75. 10.1186/s13012-022-01245-0 36309746 PMC9617234

[26] May C : Towards a general theory of implementation. *Implement Sci.* 2013;8(1): 18. 10.1186/1748-5908-8-18 23406398 PMC3602092

[27] May CR Cummings A Girling M : Using Normalization Process Theory in feasibility studies and process evaluations of complex healthcare interventions: a systematic review. *Implement Sci.* 2018;13(1): 80. 10.1186/s13012-018-0758-1 29879986 PMC5992634

[28] Damschroder LJ : Clarity out of chaos: use of theory in implementation research. *Psychiatry Res.* 2020;283: 112461. 10.1016/j.psychres.2019.06.036 31257020

[29] Murray E Treweek S Pope C : Normalisation process theory: a framework for developing, evaluating and implementing complex interventions. *BMC Med.* 2010;8: 63. 10.1186/1741-7015-8-63 20961442 PMC2978112

[30] Davidoff F Dixon-Woods M Leviton L : Demystifying theory and its use in improvement. *BMJ Qual Saf.* 2015;24(3):228–38. 10.1136/bmjqs-2014-003627 25616279 PMC4345989

[31] Yin R : Case study research and applications: design and methods.6th ed. Sage Publications,2018. Reference Source

[32] May CR Finch T Ballini L : Evaluating complex interventions and health technologies using normalization process theory: development of a simplified approach and web-enabled toolkit. *BMC Health Serv Res.* 2011;11: 245. 10.1186/1472-6963-11-245 21961827 PMC3205031

[33] Finch TL Girling M May CR : Improving the normalization of complex interventions: part 2 - validation of the NoMAD instrument for assessing implementation work based on Normalization Process Theory (NPT). *BMC Med Res Methodol.* 2018;18(1): 135. 10.1186/s12874-018-0591-x 30442094 PMC6238372

[34] CFIR Research Team: The consolidated framework for implementation research. 2022; [cited 2023 May 30]. Reference Source

[35] Bradley EH Curry LA Devers KJ : Qualitative data analysis for health services research: developing taxonomy, themes, and theory. *Health Serv Res.* 2007;42(4):1758–72. 10.1111/j.1475-6773.2006.00684.x 17286625 PMC1955280

[36] Simpson A Hannigan B Coffey M : Cross-national comparative mixed-methods case study of recovery-focused mental health care planning and co-ordination: Collaborative Care Planning Project (COCAPP). *Health Services and Delivery Research.* 2016;4(5):1–190. 10.3310/hsdr04050 26866206

[37] Mannion R Freeman T Millar R : Effective board governance of safe care: a (theoretically underpinned) cross-sectioned examination of the breadth and depth of relationships through national quantitative surveys and in-depth qualitative case studies. *Health Services and Delivery Research.* 2016;4(4):1–166. 10.3310/hsdr04040 26844311

[38] Milton R : SIMCA study material. figshare. 2024; [cited 2024Nov19]. https://figshare.com/articles/journal_contribution/SIMCA_Study_Material/27831345/1

[39] Chan AW Tetzlaff JM Gøtzsche PC : SPIRIT 2013 explanation and elaboration: guidance for protocols of clinical trials. *BMJ.* 2013;346(jan08 15): e7586. 10.1136/bmj.e7586 23303884 PMC3541470

[40] Chan AW Tetzlaff JM Altman DG : SPIRIT 2013 statement: defining standard protocol items for clinical trials. *Ann Intern Med.* 2013;158(3):200–7. 10.7326/0003-4819-158-3-201302050-00583 23295957 PMC5114123

