# Supplementary material for: The SIMCA Study Protocol: Factors influencing the implementation of the Midwifery Continuity of Carer (MCoC) model of care in NHS maternity care in England: A mixed methods cross case analysis involving clinicians, women and policy makers
Source: NIHR Open Res. 2025 Apr 24;5:4. Originally published 2025 Jan 16. [Version 2] doi: 10.3310/nihropenres.13745.2 (PMC11891474; doi:10.3310/nihropenres.13745.2)
Supplement: Peer review history

# Reviewer report: Reviewer response for version 2

**DOI:** 10.3310/nihropenres.15167.r35349

**Published:** 2025-04-29

**Authors:** Vidanka Vasilevski

**Affiliations:** 1 Deakin University, Burwood, Victoria, Australia

Thank you for giving me the opportunity to review this paper. You have addressed the queries appropriately. I wish you the best with your study.

Is the study design appropriate for the research question?

Yes

Is the rationale for, and objectives of, the study clearly described?

Yes

Are sufficient details of the methods provided to allow replication by others?

Partly

Are the datasets clearly presented in a useable and accessible format?

Not applicable

Reviewer Expertise:

Maternity service research

I confirm that I have read this submission and believe that I have an appropriate level of expertise to confirm that it is of an acceptable scientific standard.

# Reviewer report: Reviewer response for version 2

**DOI:** 10.3310/nihropenres.15167.r35351

**Published:** 2025-04-25

**Authors:** Jane Sandall

**Affiliations:** 1 Kings College London, London, London, UK

No further comments

Is the study design appropriate for the research question?

Yes

Is the rationale for, and objectives of, the study clearly described?

Yes

Are sufficient details of the methods provided to allow replication by others?

Yes

Are the datasets clearly presented in a useable and accessible format?

Not applicable

Reviewer Expertise:

maternity service delivery

I confirm that I have read this submission and believe that I have an appropriate level of expertise to confirm that it is of an acceptable scientific standard.

# Reviewer report: Reviewer response for version 1

**DOI:** 10.3310/nihropenres.14929.r34586

**Published:** 2025-03-10

**Authors:** Jane Sandall

**Affiliations:** 1 Kings College London, London, London, UK

Its important to have the overall aim consistently described in study materials. For example, The aim of the research is described differently in different study materials. The aim in the protocol is ‘To identify the local, regional and national factors which contribute to variable progress with implementation of MCoC in the NHS in England?’ In the study materials I see two aims and these need to be aligned. “to explore the factors influencing the implementation of MCoC in England, and to examine differences in how MCoC implementation has been operationalised, sustained, and experienced”.

 The design and methods are informed by implementation science frameworks in three work packages. The rational for the choice of the two frameworks guiding the Normalisation Process Theory (NPT) and the Consolidated Framework for Implementation Research (CFIR) could be explained more. A literature review that has been published. Six case studies in NHS Trusts looking at MCoC implementation. I am not sure that data analysis is a separate work package or integral to WP2. Case study site selection in WP2 is described but has not included criteria to ensure some sites have had experience of settling down implementation. All organisational change has teething problems and needs time to embed, it is hoped that some sites will fulfill the criteria.

 It is also important to interview those who have had experience of implementation. This is not clear as for example in SIMCA Board level interview guide, one question asks ‘do you believe it improves care’. Rather than asking what an individual hypothesizes, it would be more helpful to ask them of any evidence or experience they have of intended or unintended impacts on women and staff or system. Belief also implies an ideology, rather than a factual question regarding impact on care (good or bad).

 Staff surveys are notorious for poor response rates. It is hoped this is about real word experiences rather than what staff have heard from colleagues either in their own trust or elsewhere. Pre-organisational change, there is always staff anxiety, far better to hear from their actual experiences. Similarly, it is important to interview some women who have experienced MCOC. How will predicted survey response be improved, and how will women whose voices are not heard be engaged.

 This is an experienced team and the findings grounded in an implementation science framework should provide useful and relevant information for decision makers.

Is the study design appropriate for the research question?

Yes

Is the rationale for, and objectives of, the study clearly described?

Yes

Are sufficient details of the methods provided to allow replication by others?

Yes

Are the datasets clearly presented in a useable and accessible format?

Not applicable

Reviewer Expertise:

maternity service delivery

I confirm that I have read this submission and believe that I have an appropriate level of expertise to confirm that it is of an acceptable scientific standard.

# Response

**Published:** 2025-03-25

**Authors:** Rebecca Milton

**Affiliations:** Cardiff University Centre for Trials Research, Cardiff, Wales, UK

**Reviewer comment:** Its important to have the overall aim consistently described in study materials. For example, The aim of the research is described differently in different study materials. The aim in the protocol is ‘To identify the local, regional and national factors which contribute to variable progress with implementation of MCoC in the NHS in England?’ In the study materials I see two aims and these need to be aligned. “to explore the factors influencing the implementation of MCoC in England, and to examine differences in how MCoC implementation has been operationalised, sustained, and experienced”.

***Author reaponse: **Thank you for your comments. At this point the data collection is complete, and all documentation was approved by an ethics committee. Unfortunately, due to the timelines of the study this cannot be changed.*

**Reviewer comment: **The design and methods are informed by implementation science frameworks in three work packages. The rational for the choice of the two frameworks guiding the Normalisation Process Theory (NPT) and the Consolidated Framework for Implementation Research (CFIR) could be explained more.

***Author response:** The Consolidated Framework for Implementation Research (CFIR) and Normalisation Process Theory (NPT) are well-suited for studying the implementation of complex, large-scale healthcare interventions such as MCoC due to their comprehensive and process-oriented approaches.*

*CFIR provides a structured, multi-level framework to explore the key determinants of implementation, considering intervention characteristics, inner and outer settings, individuals involved, and implementation processes. This makes it particularly valuable for evaluating large-scale healthcare interventions that involve multiple stakeholders and organizational layers.*

*NPT focuses on how interventions become embedded in routine practice by analysing the social and cognitive work required for implementation. It is especially useful for understanding the dynamic processes of adoption, integration, and sustainability, making it ideal for interventions that require behavioural and cultural change within healthcare settings.*

*Together, these frameworks offer a complementary approach: CFIR helps identify barriers and facilitators to implementation at different levels, while NPT explains how and why an intervention is successfully normalised into routine practice. This combination is particularly powerful for addressing the complexities of real-world healthcare implementation beyond what other models may capture.*

**Reviewer comment:** A literature review that has been published.

***Author response:** Thank you, this has since been referenced.*

**Reviewer comment:** Six case studies in NHS Trusts looking at MCoC implementation. I am not sure that data analysis is a separate work package or integral to WP2.

***Author response:** Case study site selection in WP2 is described but has not included criteria to ensure some sites have had experience of settling down implementation. All organisational change has teething problems and needs time to embed, it is hoped that some sites will fulfill the criteria.*

*Thank you for your comment, work package 3 is a separate work package, it is a cross-case analysis and synthesis of findings of WP1 & 2.*

**Reviewer comment:** It is also important to interview those who have had experience of implementation. This is not clear as for example in SIMCA Board level interview guide, one question asks ‘do you believe it improves care’. Rather than asking what an individual hypothesizes, it would be more helpful to ask them of any evidence or experience they have of intended or unintended impacts on women and staff or system. Belief also implies an ideology, rather than a factual question regarding impact on care (good or bad).

***Author response:** Thank you for your comment, we interviewed staff and women with a range of experiences of implementation of MCoC. We take on board your feedback for future research.*

**Reviewer comment: **Staff surveys are notorious for poor response rates. It is hoped this is about real word experiences rather than what staff have heard from colleagues either in their own trust or elsewhere. Pre-organisational change, there is always staff anxiety, far better to hear from their actual experiences. Similarly, it is important to interview some women who have experienced MCOC. How will predicted survey response be improved, and how will women whose voices are not heard be engaged.

***Author response:** Thank you for your comment, NoMAd which is about real-world experiences: It is designed for measuring implementation processes from the perspective of professionals directly involved in the work of implementing complex interventions in healthcare.*

# Reviewer report: Reviewer response for version 1

**DOI:** 10.3310/nihropenres.14929.r34582

**Published:** 2025-02-21

**Authors:** Linda Sweet

**Affiliations:** 1 Deakin University Geelong, Victoria, Australia

Thank you for the opportunity to review this study protocol. It is a timely and much-needed study. The team is strong, and the work packages are achievable. 

 There are a few acronyms used before being qualified and some minor anthropomorphic statements such as 'This study proposes .."

 In WP2, the first sentence for study participants is confusing at first read and is one very long sentence.

 I look forward to following the outcome of this work.

Is the study design appropriate for the research question?

Yes

Is the rationale for, and objectives of, the study clearly described?

Yes

Are sufficient details of the methods provided to allow replication by others?

Yes

Are the datasets clearly presented in a useable and accessible format?

Not applicable

Reviewer Expertise:

Midwifery

I confirm that I have read this submission and believe that I have an appropriate level of expertise to confirm that it is of an acceptable scientific standard.

# Response

**Published:** 2025-03-25

**Authors:** Rebecca Milton

**Affiliations:** Cardiff University Centre for Trials Research, Cardiff, Wales, UK

**Reviewer comment:** There are a few acronyms used before being qualified and some minor anthropomorphic statements such as 'This study proposes .."

***Author response: **Thank you for your comment about the acronyms, this has been amended. Regarding your comment about use of anthropomorphic statements I have edited this specific example to the following: The aim of the study is to… and similarly “the study aims…” has been changed to “the aim of the study”*

**Reviewer comment:** In WP2, the first sentence for study participants is confusing at first read and is one very long sentence. I look forward to following the outcome of this work.

***Author response:*** *Thank you this has been restructured.*

# Reviewer report: Reviewer response for version 1

**DOI:** 10.3310/nihropenres.14929.r34730

**Published:** 2025-02-17

**Authors:** Vidanka Vasilevski

**Affiliations:** 1 Deakin University, Burwood, Victoria, Australia

Thanks for inviting me to review this paper. It is well established that MCoC improves a range of outcomes for women, babies, and the healthcare system, however, successful implementation remains a significant challenge. Understanding MCoC implementation, and the various factors that influence it, will contribute knowledge that can support broader implementation of MCoC internationally. Overall, this is a very clear and well written protocol, however a bit more detail is required in the methods. Please see my  queries related to specific sections of the paper below:

 Abstract: Nice summary of the overall study

 Introduction: Great introduction, covering background and rationale to the study. A minor point, generally you would not start a sentence with an acronym, but with the full term.

 Theoretical/conceptual framework: The following sentence 'CFIR is not intended to be applied wholesale...' I don't think 'wholesale' is the right term here.

 WP2:

 Study setting: Could more details about the NHS settings in England be provided, are they from areas with similar demographics or are they diverse?

 Study participants: '15 interviews per site (n=10)' The (n=10) placements appears as if you are including 10 sites, possibly move it after managers, midwives etc., and then (n=5) after women enrolled in MCoC.

 How many participants are you aiming for the surveys? Consider sample size calculation if appropriate.

 Data collection and management: What is 'Speak Up Guardians' please define for the non-UK audience.

 Please describe the tools you are using in more detail, are they validated tools or tools designed specifically for this project?

 You state that components will be summarised and examined for potential associations, what outcomes will you be looking for specifically?  

 Recorded semi-structured interviews in six case study sites: n=90? Stated above n=65, please clarify. The detail here is also repetitive of the above, decide where it fits better.

 Data analysis:

 There is no mention of what statistical tests are anticipated to be used for the survey component.

 Overall: There are many acronyms used throughout, which impact the flow of reading when they are not familiar, consider minimising the number of acronyms used.

Is the study design appropriate for the research question?

Yes

Is the rationale for, and objectives of, the study clearly described?

Yes

Are sufficient details of the methods provided to allow replication by others?

Partly

Are the datasets clearly presented in a useable and accessible format?

Not applicable

Reviewer Expertise:

Maternity service research

I confirm that I have read this submission and believe that I have an appropriate level of expertise to confirm that it is of an acceptable scientific standard, however I have significant reservations, as outlined above.

# Response

**Published:** 2025-03-25

**Authors:** Rebecca Milton

**Affiliations:** Cardiff University Centre for Trials Research, Cardiff, Wales, UK

Reviewer comment: Abstract: Nice summary of the overall study. Introduction: Great introduction, covering background and rationale to the study. A minor point, generally you would not start a sentence with an acronym, but with the full term.

*Author response: Thank you for your comment, I can confirm that this has been amended. NHS has been replaced with National Health Service (NHS).*

 Reviewer comment: Theoretical/conceptual framework: The following sentence 'CFIR is not intended to be applied wholesale...' I don't think 'wholesale' is the right term here.

*Author response:* *Thank you for your comment, the wording has been amended to: CFIR offers numerous constructs to consider when investigating implementation of complex interventions and will be applied accordingly.*

 Reviewer comment: WP2: Study setting: Could more details about the NHS settings in England be provided, are they from areas with similar demographics or are they diverse?

*Author response: Thank you for your query, the study sites are to remain anonymous. However, I feel that this point is addressed in: ‘Sampling and selecting case studies’. For example, we have described how geographical and organisational diversity will be considered during site sampling. We have also acknowledged the consideration of different demographics in the rewording as follows "Consideration of geographical settings of case study sites to ensure that case studies reflect, where possible, demographic and regional differences in rural, urban, and inner-city areas of England."*

 Reviewer comment: Study participants: '15 interviews per site (n=10)' The (n=10) placements appears as if you are including 10 sites, possibly move it after managers, midwives etc., and then (n=5) after women enrolled in MCoC. How many participants are you aiming for the surveys? Consider sample size calculation if appropriate.

*Author response: Thank you for your comment, I agree with your point about the way this sentence is structured: 15 interviews per site (n=10) purposively sampled participants including those directly involved in MCoC implementation, for example, managers, midwives, obstetricians and (n=5) women enrolled in MCoC. This has been altered to 15 interviews per site, consisting of purposively sampled participants including those directly involved in MCoC implementation, for example, managers, midwives, obstetricians (n=10) and women enrolled in MCoC (n=5). Regarding the anonymous survey we did not have a target number for completion, this wasn’t something we could monitor. We have retrospective numbers now. In study set up it was advised that the survey responses were not included in the site sample sizes.*

 Reviewer comment: Data collection and management: What is 'Speak Up Guardians' please define for the non-UK audience.

*Author response: Thank you for your comment, this has been changed to Staff Speak Up. The aim of this intervention is to help the NHS deliver the People Promise for workers; by ensuring they have a voice that counts and by developing a speaking up culture.*

 Reviewer comment: Data collection and management: Please describe the tools you are using in more detail, are they validated tools or tools designed specifically for this project?

*Author response: Thank you for your comment, the text has been updated to: Staff survey: a validated staff survey tool (NOMAD) ^33^ will be used to collect the perceptions and experiences of maternity staff about the implementation of MCoC in the maternity services within which they work.*

 Reviewer comment: Data collection and management: You state that components will be summarised and examined for potential associations, what outcomes will you be looking for specifically?  

*Author response: Thank you for your comment. To clarify, the data generated does not aim to analyse outcomes or associations between outcomes. The survey instruments measure implementation experience and perceptions, not outcomes, enabling possible inference between roles and organisational characteristics as described in the text*

 Reviewer comment: Data collection and management: Recorded semi-structured interviews in six case study sites: n=90? Stated above n=65, please clarify. The detail here is also repetitive of the above, decide where it fits better.

*Author response: Thank you for your query, there are two sets of interviews being conducted. One is with national and regional leads (n=65) and the second is with study participants (n=90). This section has been restructured to improve readability.*

 Reviewer comment: Data analysis: There is no mention of what statistical tests are anticipated to be used for the survey component.

*Author response:* *We state that ‘Cronbach α testing will be conducted on all four NPT components, to measure the internal consistency of the constructs within the context of this study. Each NPT component will be derived as the mean score of the four questions in the survey that correspond to that NPT component. Components will then be summarised and examined for potential associations by various roles or organisational characteristics. Descriptive statistics and bar charts will help visualise the ‘shape’ of the data within and eventually across case sites. These steps will help identify interesting or anomalous features within the data and prove useful in then generating cross-tabulations and scattergrams of the relationships between implementation factors and other variables. Survey analysis will be undertaken via SPSS.’ This has been moved to the analysis section.*

 Reviewer comment: Overall: There are many acronyms used throughout, which impact the flow of reading when they are not familiar, consider minimising the number of acronyms used.

*Author response:* *Thank you, I have reviewed this and have removed the less frequently used acronyms throughout.*
